# Liquid biopsies for multiple myeloma in a time of precision medicine

**DOI:** 10.1007/s00109-020-01897-9

**Published:** 2020-04-04

**Authors:** Bruna Ferreira, Joana Caetano, Filipa Barahona, Raquel Lopes, Emilie Carneiro, Bruno Costa-Silva, Cristina João

**Affiliations:** 1 Myeloma and Lymphoma Research Programme, Champalimaud Centre for the Unknown, Lisbon, Portugal; 2 Hemato-Oncology Unit, Myeloma and Lymphoma Research Programme, Champalimaud Centre for the Unknown, Lisbon, Portugal; 3 Systems Oncology Group, Champalimaud Centre for the Unknown, Lisbon, Portugal; 4 Hemato-Oncology Unit, Myeloma and Lymphoma Research Programme, Nova Medical School, Champalimaud Centre for the Unknown, Lisbon, Portugal

**Keywords:** Multiple myeloma, Liquid biopsy, Biomarkers, Precision medicine

## Abstract

Multiple myeloma (MM) is a challenging, progressive, and highly heterogeneous hematological malignancy. MM is characterized by multifocal proliferation of neoplastic plasma cells in the bone marrow (BM) and sometimes in extramedullary organs. Despite the availability of novel drugs and the longer median overall survival, some patients survive more than 10 years while others die rapidly. This heterogeneity is mainly driven by biological characteristics of MM cells, including genetic abnormalities. Disease progressions are mainly due to the inability of drugs to overcome refractory disease and inevitable drug-resistant relapse. In clinical practice, a bone marrow biopsy, mostly performed in one site, is still used to access the genetics of MM. However, BM biopsy use is limited by its invasive nature and by often not accurately reflecting the mutational profile of MM. Recent insights into the genetic landscape of MM provide a valuable opportunity to implement precision medicine approaches aiming to enable better patient profiling and selection of targeted therapies. In this review, we explore the use of the emerging field of liquid biopsies in myeloma patients considering current unmet medical needs, such as assessing the dynamic mutational landscape of myeloma, early predictors of treatment response, and a less invasive response monitoring.

## Introduction

Multiple myeloma (MM) is a plasma-cell malignancy characterized by bone lesions that is virtually always preceded by a monoclonal gammopathy of undetermined significance (MGUS) [1, 2]. The diagnosis of multiple myeloma is based on the presence of clinical, biochemical, histopathological, and radiological markers of disease. Biological characteristics of MM as well as patient- and drug-dependent factors, such as health status of the patient and treatment toxicities, dramatically influence survival [3, 4].

To address MM clinical heterogeneity, scoring systems have been developed in order to estimate individual prognosis. The degree of anemia, renal failure, and osteolysis were the first disease-related prognostic biomarkers, included in the Salmon & Durie (SD) staging system. Subsequently, serum albumin and β2-microglobulin levels were incorporated in the International Staging System (ISS), reflecting patient tumor burden, turnover rate, presence of renal impairment, and nutritional and performance status [5]. The prognostic performance of the ISS score was updated by adding high-risk cytogenetics [t(4;14), t(14;16), and del17p determined by interphase fluorescence in situ hybridization] and elevated serum lactate dehydrogenase [6]. More recent, deletions and amplification of chromosome 1 were added as conferring worse prognosis [7–9]. Currently, R-ISS is used primarily for risk stratification of patients with clinical implications with regard to selection of therapy but not in a generalized way.

To determine osteolysis and bone marrow involvement, imaging techniques are currently used. The European Myeloma Network and the European Society for Medical Oncology guidelines have recommended whole-body low-dose computer tomography (WBLDCT) as the imaging modality of choice for the assessment of MM-related lytic bone lesions. Magnetic resonance imaging is the gold-standard imaging modality for detection of bone marrow involvement, whereas positron emission tomography/computed tomography (PET/CT) provides valuable prognostic data and is the preferred technique to assess response to therapy [10].

In clinical practice, bone marrow (BM) biopsy, mostly performed in one site, is used to access the genetic profile of the disease in MM patients. However, tissue biopsies fail to capture the intratumoral and inter-metastatic genetic heterogeneity, which decrease the accuracy of tests based on them [11]. MM is probably not a single entity and comprises a number of molecular subgroups characterized by a compilation of genomic alterations [12, 13]. Therefore, considering the MM clonal heterogeneity, BM biopsies probably do not reflect the true mutational profile in MM due to sampling bias. Also, BM biopsy’s invasive nature hinders serial clonal monitoring. Accurately measuring tumor burden is also crucial for prognostication. The most recent consensus statement from the IMWG regarding BM PC estimation requires either BM aspiration and/or biopsy However, PC counts on BM aspirates by conventional morphologic or immunohistochemical analysis may vary significantly due to dilution with peripheral blood and the patchy pattern of PCs infiltration. The development of new markers and approaches to more accurately and quickly assess tumor burden in MM patients would result in better outcomes.

The emergence of several new drugs over the past decades has dramatically improved patient outcomes in MM, extending the median survival by 4 years [4, 14]. Complete response (CR) rates have increased in parallel, establishing the need to develop more sensitive methods to better define depth of response as well as to monitor minimal residual disease (MRD) over time. Measurement of MRD in bone marrow by both next-generation sequencing (NGS) of variable diversity joining V(D)J rearrangements or next-generation flow cytometry (NGF) is highly predictive of survival in MM [15, 16] and may be used as a biomarker to adapt treatment strategies [17, 18]. However, serial assessments of MRD involve repeated sampling, which imposes the trauma of repeated BM aspirations. Furthermore, false negative MRD values may be obtained due to BM dilution with blood and sampling bias related with the patchy distribution of clonal plasma cells. A way of capturing tumor heterogeneity and potentially decide upon treatment over the course of time in a minimal invasive method in MM patients may be the use of liquid biopsies.

Liquid biopsies include the sampling and testing of biological fluids, typically blood, for a subset of circulating tumor components. The tumor components that can be tested include, among others, circulating tumor cells (CTC), tumor nucleic acids (such as circulating tumor DNA (ctDNA) and microRNA), and extracellular vesicles (EV). This “tumor circulome” can be used as a source of biomarkers for cancer diagnosis, screening, and prognosis [19]. The FDA already approved liquid biopsies for several cancers. In 2016, liquid biopsies were approved for lung cancer prognostication and for colorectal cancer based on ctDNA content [20, 21]. In MM, liquid biopsies are currently being evaluated in clinical studies such as the Liquid Biopsy Evaluation and Repository Development at Princess Margaret study (NCT03702309), The MMRF Cure Cloud Multiple Myeloma Research Initiative (NCT03657251), and the Study to Assess for Measurable Residual Disease (MRD) in multiple myeloma patients (NCT04108624).

Using new biomarkers to improve prognostic models in MM will certainly lead to the development of risk-adaptive therapeutic strategies improving the outcome of this disease and paving the way towards precision therapy.

## Precision medicine in multiple myeloma

In MM, genome studies have led to a better understanding of the disease, including its genetic heterogeneity, evolution patterns over time, and identification of potential molecular drivers [22]. This complex molecular biology of MM has been described in several studies [23–29] and includes the observation of dynamic intra-patient sub-clonal heterogeneity [23, 25, 27, 30–34] and appearance of distinct sub-clones along longitudinal sampling [30, 32, 33]. Nevertheless, some genes are recurrently mutated in MM. Mutations affecting the RAS/MAPK pathway, such as *KRAS*, *NRAS*, and *BRAF*, were found to be the most frequently observed pathway mutations in MM detected in approximately 40% of patients [12]. Interestingly, 4–9% of MM patients harbor BRAF mutation at diagnosis, with the BRAF V600E mutation being the most common, with even higher frequency at relapse (up to 18%) [23, 24]. Although preclinical studies have shown the cytotoxic effect of BRAF inhibitors in MM cell lines [35], the evidence on using precision medicine approaches to BRAF mutational status and the efficacy of BRAF inhibitors is scarce [36–38]. Another example of potential use of precision medicine in MM is targeting the Bcl-2 apoptotic pathway. MM cell lines and patient samples, particularly those with t(11:14), have been shown to be particularly sensitive to Bcl-2 inhibitors, making Bcl-2 a potential target in this subtype of myeloma. Although the presence of the t (11:14) is used to be considered a standard risk factor, it is increasingly thought of as an intermediate risk factor in the era of novel agents, conferring worse outcome compared with standard-risk myeloma. Venetoclax is an oral compound designed to specifically inhibit the Bcl-2 protein in cancer cells. The sensitivity to the drug has been correlated to the ratio of Bcl-2 to Mcl-1 and Bcl-X_L,_ with high Mcl-1 levels conferring resistance to this drug [39]. In the MM clinical setting, venetoclax has been shown to be well tolerated and effective in different phase I/II trials [40–43]. In order to better determine efficacy, a phase III trial comparing venetoclax in combination with standard treatment bortezomib-dexamethasone (BELLINI trial—M14-031; NCT02755597) is ongoing. However, in March 2019, the trial was interrupted due to safety concerns related to the higher rate of patient deaths with venetoclax combined with bortezomib (Velcade) and dexamethasone (Vd) (Ven + Bd) compared to placebo plus Vd in patients with relapsed/refractory myeloma. However, patients with t [11, 14] had consistent clinical benefit when treated with Ven + Bd, and a biomarker-driven approach with venetoclax seems appropriate in MM.

The new insight into the genetic landscape of MM provides a valuable opportunity for the implementation of precision medicine approaches, enabling patient profiling and selection of potential targeted therapies. However, there are still many challenges for precision medicine in MM, primarily because there is no unique driver mutation in MM and therefore the design of a selective targeted therapy is unlikely to benefit all patients. On the other hand, assessing the disease complexity at diagnosis and over time using liquid biopsies may provide a less invasive method for dynamic disease monitoring and therapeutic guidance. There would be several advantages to this approach guiding therapy, accelerating therapeutic switch to more affective alternatives, avoiding unnecessary side effects of ineffective treatments, and optimizing dose adjustments.

Here, the potential clinical applications of liquid biopsies are discussed, including CTC, ctDNA, microRNA (miRNA), and extracellular vesicles in MM.

### Circulating tumor cells

In MM, CTCs are released from the primary tumor into the bloodstream [44], homing again to the BM at different locations in a metastatic dissemination process [45]. Migration of CTCs seems to be an early event in carcinogenesis [46] and since they were first described in 1869 by Ashworth, several studies have provided evidence of their presence in cancer patients [47, 48]. In recent years, they have gained increasing importance because they are minimally invasive indicators that can reveal critical information about the tumor. Indeed, CTCs may have numerous clinical applications, namely in detection, characterization, treatment guidance, and follow-up of cancer patients [49].

Several novel techniques to detect CTCs have emerged, either through nucleic acid-based or cytometric methods [50–53]. The first approach is highly sensitive and relies on the detection of specific DNA or RNA sequences expressed by tumor cells. However, cytometric assays have been proved to be easier to implement and have been privileged to identify and characterize CTCs according to their immune profile, size, and expression of specific markers [47]. Currently, CellSearch is the only FDA-approved technology for extraction and enumeration of CTCs of epithelial origin in the whole blood in specific cohorts of patients with solid cancers, namely breast, prostate, and colorectal carcinomas [54–57]. Despite the low number of cells and morphologic heterogeneity, this system is able to provide an accurate, sensitive, and reproducible way to count CTCs [58]. Still, this is not straightforward for all types of cancer and further development is needed to expand this approach to other neoplasias, such as MM.

Several research groups have tried to improve the tools to detect and characterize CTCs in MM. Foulk and colleagues were able to develop a kit for enumeration and characterization of CTCs by fluorescence in situ hybridization (FISH) and next-generation sequencing (NGS), showing more CTCs in all stages of MM compared to healthy donors. They showed that the number of CTCs in newly diagnosed myeloma patients correlates with other disease characteristics, such as the percentage of PCs in the BM, serum M protein, and International Staging System (ISS) [59]. In another study, Zhang and his team developed a linear and accurate cell-based immunofluorescence assay to distinguish MM CTCs from normal leukocytes based on specific morphological parameters and expression levels of CD138 and CD45 [60].

In a study analyzing the transcriptional profile of CTCs in MM, the gene expression profile of those cells was found to be identical to BM clonal PCs. However, CTCs were shown to have a higher expression of genes involved in key functions, such as inflammatory response, hypoxia, cell cycle, and migration, some of which associated with significantly inferior progression free survival and related to more aggressive disease [61]. Another study reported similar findings at a single-cell level, showing that in half of the patients, the proportion of mutated CTCs was significantly higher than in single cells from BM samples [62]. This suggests that CTCs may provide relevant information regarding sub-clonal assessment in MM, beyond what BM findings show.

Several research groups have been successful in developing new methods to detect CTCs, correlating their numbers and characteristics with an unfavorable prognosis [35, 63, 64]. The number of CTCs was found to be a predictor of survival in patients with both newly diagnosed [65] and relapsed disease [66]. Interestedly, high levels of CTCs in smoldering MM (SMM) were associated with a high risk of progression to overt MM in the first 2–3 years after diagnosis [67]. Recently, using next-generation flow (NGF) method developed by EuroFlow, Sanoja-Flores et al. showed that CTCs in PB at diagnosis are associated with poorer outcome of both MGUS and MM patients [15].

### Cell-free circulating tumor DNA

Noninvasive assessment of tumor DNA is possible with cell-free circulating tumor DNA as well as from circulating tumor cells. In contrast to the circulating intact tumor cells in blood**,** ctDNA consists of small fragments of nucleic acids extracted from the plasma or serum. The cancer-derived fragments may be identified if they contain tumor-specific mutations or other genetic aberrations. Both quantitative and qualitative information can be obtained from ctDNA analysis. Due to the short half-life of DNA (approx. 2,5 h), ctDNA quantification provides a real-time snapshot of tumor bulk. Qualitative information stems from the description of genetic alterations found in ctDNA, which may be assessed overtime to show clonal evolution in MM. [68]

Deep sequencing of ctDNA from MM patients is a technique with a high sensitivity and has recently been demonstrated to recapitulate mutational profiles of matched BM aspirates [69]. Analysis of plasma-derived ctDNA as an adjunct to BM biopsy, for mutational characterization and tracking disease progression, is currently possible using droplet digital PCR [70]. Kis et al. reported that sequencing of ctDNA enables the analysis of sub-clonal hierarchies, reflecting tumor profiling with high levels of concordance with matched BM samples [71]. However, in some cases, the mutations identified were found only in plasma, which is consistent with the spatial heterogeneity of MM previously demonstrated by multi-region DNA sequencing of BM plasma cells [72–74]. In that sense, incorporating plasma ctDNA evaluation aimed at identifying frequent mutations found in MM may represent a significant advance in attempts to personalize MM treatment strategies. Apparently, the assessment of ctDNA at diagnosis allows the identification of clonotypes, confirming the ability of ctDNA to provide an alternative noninvasive test when the disease is active and guide the study of intraclonal heterogeneity and possibly drug choice.

Because of easy accessibility, ctDNA sampling is suitable for repeated analyses and, as it correlates with disease progression, it could serve as a prognostic marker as well. This was shown for acute leukemia and for both Hodgkin and non-Hodgkin lymphomas [73, 74] but not yet for MM. Historically, in 1977, Leon et al. described significantly elevated levels of ctDNA in PB of patients with various solid malignancies (lung, kidney, prostate, and ovarian cancers) in comparison with healthy donors (HD). The average concentration of ctDNA was about 13 ng/mL for HD, while in cancer patients, ctDNA reached levels up to 5000 ng/mL, in patients with advanced metastatic disease. Furthermore, after radiotherapy, the levels of ctDNA decreased in most of the patients and correlated with pain and tumor size reduction. On the other hand, persistently elevated levels of ctDNA were associated with resistance to treatment and poor prognosis [75]. In MM, ctDNA may be used to monitoring disease by next-generation sequencing of V(D)J rearrangements and for detecting minimal residual disease. [76, 77]

ctDNA has been considered as a promising noninvasive tool for monitoring response to treatment, particularly in situations of active disease. However, given that ctDNA may be undetectable in more than half of the patients with positive MRD in the BM [76, 78], it may not yet serve as a robust biomarker for disease monitoring compared to NGS or NGF. In the study by Oberle et al. [76], only 39% of patients with less than a very good partial response displayed detectable ctDNA, suggesting that the mechanisms by which M protein and ctDNA are released into the bloodstream appear to be independent of each other. Thus, monitoring the disease using ctDNA may be a possibility in situations where M protein is not a reliable biomarker, such as in light chain escape and non-secretory or oligo-secretory disease. Moreover, qualitative information can be obtained by examining the genetic alterations associated with the tumor, facilitating decision-making based on precision medicine [79].

### Circulating microRNA

MiRNAs are small sequences of RNA, approximately 25 nucleotides long, found in various body fluids [80, 81]. In blood, miRNAs circulate stably bound to proteins, high-density lipoproteins, within extracellular vesicles such as exosomes or in apoptotic vesicles [82, 83]. They regulate gene expression by either promoting messenger RNA (mRNA) degradation or repressing its translation, playing crucial roles in a variety of physiological- and cancer-related processes, including cell motility, differentiation, proliferation, and apoptosis [84]. It is not yet certain whether circulating miRNAs are passively released into the circulation from apoptotic and necrotic cells or if they are specifically secreted, for instance, in exosomes [85, 86].

Methods for measuring circulating miRNAs are not yet validated or standardized. Current strategies begin with extraction and purification of circulating miRNAs from plasma or serum, followed by screening and identification of sequences of interest using micro-arrays, subsequently confirmed and validated by RT-PCR. NGS, although potentially useful for discovering new miRNAs, is more expensive and time-consuming and needs further development for this application.

Several groups have identified different miRNA with important roles in MM pathogenesis and progression, providing a potential tool for distinguishing MM patients from healthy controls and MGUS patients (Table 1). In MM, miRNAs exhibited similar expression patterns in peripheral blood and BM aspirates [87], which makes them good candidates for biomarkers of disease both at diagnosis, during treatment, and relapse.

**Table 1:** Overview of specific miRNA as potential biomarkers for MM and other monoclonal gammopathies

Study design	Upregulated	Downregulated	Reference
MM patients vs healthy controls	miR-142-5p	miR-17	[88–93]
	miR-29a	miR-19a	
	miR-660	miR-19b	
	miR-202	miR-20a	
	miR-148	miR-92a	
	miR-181a	miR-1308	
	miR-20a	miR-191	
	miR-221	miR-130a	
	miR-99b	let-7d	
	miR-146a	miR-103	
	miR-16	let-7e	
	miR-186	miR-744	
	miR-454	miR-151-5p	
	miR-483-5p		
	miR-720		
	miR-1246		
	miR-218		
	miR-34a		
	miR-1274A		
	miR-138		
	miR-10b		
	miR-1243		
Newly diagnosed MM patients vs healthy controls	miR-135b-5p	miR-19a	[94, 95]
	miR-214-3p miR-33b	miR-92a miR-20a	
	miR-3658		
	miR-4254		
	miR-483-5p		
MGUS vs healthy controls	miR720	miR-19a	[91–93, 96]
	miR-1246	miR-1308	
	miR-34a	miR-744	
		miR-130a	
		let-7d	
		let-7e	
		miR-16	
		miR-25	
		miR-20a	
		miR-25	
		miR-660	
MGUS vs MM patients	miR-19a		[91, 96]
	miR-25		
MM patients at relapse vs diagnosis	miR-34a	let-7e	[93]
MM patients at diagnosis vs patients in complete response	–	miR-16	[96]
		miR-25	
		miR-20a	
		miR-25	
		miR-660	

Thus far, no single miRNA was shown to predict the evolution from MGUS to MM. However, the combination of miR-1246 and miR-1308 was able to distinguish between MGUS and MM [92]. Also, in MGUS patients, miR-92a plasma level was significantly higher compared to MM patients, but there was no significant difference between patients with smoldering disease and MGUS [91].

MiRNAs have been reported as a prognostic tool and correlate with survival outcomes in MM [80, 92–94, 96, 97]. Concerning the response to treatment, miR-92a plasma levels were described to return to baseline in patients achieving CR but not in those achieving only partial response (PR) or very good partial response (VGPR) [91]. Patients with high serum expression levels of miR-17-92 cluster had shorter PFS compared to those with low level expression [98], suggesting that this cluster is associated with poor prognosis in MM. Low level miR-483-5p was associated with better PFS [95]. Multivariate analysis revealed that miR-19a was a significant predictor of shorter PFS and OS [94]. Expression of some miRNAs dynamically change in MM patient’s plasma/serum during disease progression, and so continuous detection of miRNA levels in blood could be used to monitor disease status and assess prognosis. High miR-34a and low let-7d expressions were observed in relapsed MM patients compared to levels in patients at the time of diagnosis [91].

There seems to be a relation between changes in miRNA pattern of expression and specific genetic abnormalities, particularly cytogenetic abnormalities associated with poor prognosis such as del(13q14), 1q12 amplification, or t(4;14) [93]. Deregulation of target genes of miRNAs upregulated in t(4;14) MM genetic subtype seems to promote oncogenesis by modulating the expression of proteins associated with cellular growth and proliferation (Table 2).

**Table 2:** Correlation of miRNA and cytogenetic abnormalities

miRNA	Upregulation/Downregulation	Cytogenetic abnormalities	Reference
miR-19a	Downregulation	del(13q14) and 1q21 amplification	[94]
miR-99b	Upregulation	t(4;14)	[90]
miR-211	Downregulation	del(13q)	[90]
let-7e and miR-744	Downregulation	del(13q)	[93]
miR-744	Downregulation	1q12 amplification or t(4;14)	[93]
miR-15 and miR-16	Downregulation/ Loss	del(13q14)	[99]

As an example, the gene for miR-744 is in the 17p12 region where various tumor-related genes are closely situated (TP53, BRCA1, and FBXO4) and deletions at chromosome 17p13.1-17p12 were previously associated with poor survival in cancers [100]. Moreover, downregulation of several miRNAs resulted in overexpression of cyclin D2 (CCND2) as observed in t(4;14) and t(14;16), suggesting for the first time that miRNA expression patterns in MM were correlated with protein expression patterns in specific genetic abnormalities [101].

Furthermore, targeting deregulated miRNA in MM might be a promising therapeutic approach. Among the potential targets, miR-21 is upregulated in MGUS and MM, promoting survival and progression. Interleukin-6 is responsible for regulating miR-21 through Stat-3 activation, a central pathway for MM cell growth and drug resistance. Tumor suppressor genes such as PTEN, BTG2, and Rho-B are targeted by miR-21. Upregulation of their expression with oligonucleotide inhibitors of miR-21 may result in anti-tumor activity against MM [102].

Additionally, aberrant expression of several miRNAs has been observed in drug-resistant myeloma cell lines, suggesting that deregulated miRNAs might be involved in drug resistance of MM cells (Table 3).

**Table 3:** Correlation between miRNA and drug resistance in MM

miRNA	Upregulation/downregulation	Treatment	Indicator	Reference
miR-15a and miR-16-1	Upregulation	Cytotoxic agents	Increased growth and survival of MM cells	[94]
miR-19a	Downregulation	Bortezomib	Improved PFS and OS	[94]
miR-202	Upregulation	Bortezomib	Increased sensitivity	[103]
miR-513a-5p, miR-20b-3p, let-7d-3p	Upregulation	Bortezomib	Increased resistance	[104]
miR-125b-5p, miR-19a-3p, miR-21-5p, miR-20a-5p, miR-17-5p, miR-15a-5p, miR-16-5p	Downregulation	Bortezomib	Increased resistance	[104]
miR-19b and miR-331	Upregulation	ASCT	Longer PFS	[96]
miR-483-5p	Downregulation	TAD, VC, PAD, TD and VTD	Higher PFS	[95]
miR-26a-5p, miR-29c-3p, miR-30b-5p, miR-30c-5p, miR-193a5p, miR-331-3p,	Downregulation	Lenalidomide with low dose Dexamethasone	Shorter TTP	[105]

### Extracellular vesicles

Extracellular vesicles (EVs) are bilayer lipid particles naturally released from all cells [106]. Major roles for EVs have been proposed in numerous physiological and pathological processes. In cancer, EVs are emerging as novel players in intercellular communication that transfer cargo molecules (including RNA, DNA, proteins, among others) that, when up taken by target cells, can influence their behavior [107]. EVs derived both from MM cells and bone marrow stromal cells (BMSC) have been found to intervene in key processes of MM such as tumor progression [108], immunosuppression [109], osteogenesis [110, 111], angiogenesis [112–114] procoagulant activity [115], and drug resistance [116–118] through the transfer of proteins and regulation of miRNA expression in the bone marrow microenvironment.

Since EVs can be found in several biological fluids, such as peripheral blood [119], it makes them an attractive biomarker in liquid biopsies. In the clinical setting, we and others have shown that MM patients have different EV miRNA expression levels and protein content compared to healthy subjects [116, 120]. Moreover, the content analysis of these EVs has been used as prognostic factor [116] and to predict therapy resistance in MM patients [117].

Because EVs are a new field of research, several challenges remain, including the nomenclature of distinct subtypes of EVs, the lack of good established markers, and diversity of separation protocols [107]. In an attempt to improve standardization to the field, the International Society for Extracellular Vesicles recently updated their guidelines for the analysis of EVs and the reporting of the results. For instance, special considerations for EV separation from biological fluids such as blood derivates need to be considered and technical factors should be recorded for reproducibility. Factors as such as donor age, biological sex, diet, specific diseases, and medications, among many others, may affect circulating EV [106]. On the other hand, technical factors including pre-analytical variables such as source of EV, storage conditions, manipulation of the source material, and experimental conditions can affect EV recovery [106].

Finally, one should take into account that, according to these guidelines, complete isolation of EVs from other entities is currently an unrealistic goal. Separation of EVs from other non-EV components can be achieved to various degrees by the different techniques. The degree of EVs purification depends on the experimental question and the need to attribute a function to vesicles as compared with other particles [106].

Precise characterization of RNA, DNA, and protein components of EVs and non-vesicle compartments are needed to clarify the heterogeneity of EVs. This is crucial to identify biomarkers and avoid potential overlap when using such components as liquid biopsies. Recently, Jeppesen et al. demonstrated that extracellular double-stranded DNA (dsDNA) and histones are not associated with exosomes or any EVs but instead released as non-vesicular entities, proposing a new model for active secretion of extracellular DNA through autophagy and multi-vesicular-endosome–dependent but exosome-independent mechanism [121].

In MM, several studies have investigated EVs as an active vehicle for molecules that can modulate the BM microenvironment. In 2013, Roccaro et al. demonstrated that EVs derived from BM mesenchymal stromal cells (BM-MSCs) of MM patients have different functional activity compared to healthy donors, showing higher contents of oncogenic proteins, cytokines, and proteins that are regulators of adhesion and migration [108]. Considering bone disease, Faict et al. demonstrated in vivo that MM-derived EVs could induce osteolysis in a similar pattern as the MM cells themselves [110]. Interestingly, the authors showed that blocking EVs secretion using the sphingomyelinase inhibitor GW4869 not only increased cortical bone volume but also sensitized the myeloma cells to bortezomib, leading to a stronger anti-tumor response when GW4869 and bortezomib were combined. On the other hand, Zarfati el al. showed that EVs released from MM cell lines after treatment with bortezomib promoted suppression of angiogenesis by decreasing proliferation and migration of endothelial cells [114], suggesting that EV-mediated cell to cell communication in MM BM microenvironment may influence mechanisms of drug resistance.

In the clinical setting, Manier et al. showed that the use of miRNAs contained in circulating EVs predict poor prognosis in newly diagnosed MM patients [116]. These authors analyzed EVs isolated from serum samples from 156 patients with newly diagnosed MM uniformly treated with bortezomib and dexamethasone and showed that let-7b and miR-18a were significantly associated with poor PFS and overall survival (OS), independently of the International Staging System and cytogenetics. Moreover, Zhang et al. showed that a downregulation of exosomal miR-16-6p, miR-15a-5p, miR-20a-5p, and miR-17-5p was found in patients resistant to bortezomib [117]. In another study, Sun et al. reported that EV miR-214 secreted by osteoclasts is transferred to osteoblasts, inhibiting their activity. Circulating miR-214 levels were found to be significantly increased in EVs from osteoporotic patients in comparison to the non-osteoporotic ones, suggesting a potential use of EVs as new biomarkers for MM-related bone disease.

Since MM is a multifocal disease with spatial and genetic heterogeneity, one can hypothesize that the cellular crosstalk through EVs can be implicated in disease behavior, sustaining potential transfer of mechanisms of progression or drug resistance between cells in different niches. In the future, by having access to circulating EVs in MM patients, it will be possible to have a new and dynamic insight into this disease, in a minimally invasive way.

## Discussion and conclusions

Tumor biopsy is currently the gold standard for assessing somatic alterations, but this approach is invasive and does not consider tumor heterogeneity. Liquid biopsies may be considered a better alternative because of their noninvasive, rapid, precise, and almost real-time attributes. However, before being applied to the clinical setting, these methodologies need to be harmonized and validated in well-powered and well-designed studies. One of the primary prerequisites is the incorporation of these analyses in the follow-up strategy and checking of the concordance with gold-standard detection methods as imaging, M protein concentration, and biopsy histology.

In this review, an overview of the use of the emerging new field of liquid biopsies in myeloma patients is provided considering current unmet medical needs such as better access to the dynamic mutational landscape of myeloma, early predictors of treatment response, and disease monitoring overtime in a less invasive way. These biomarkers add to the recent insights into the genetic landscape of MM, providing a valuable opportunity to implement precision medicine approaches with the objective of enabling better patient profiling and selection of targeted therapies. Table 4 summarizes some of the advantages and limitations of the different approaches here analyzed.

**Table 4:** Comparison between the different approaches to liquid biopsies

	Advantages	Limitations
CTCs	-Several methods available for enumeration and characterization of CTCs (PCR, flow cytometry, image-based immunomagnetic, microchip) -Potential to consistently enumerate, track, and characterize CTCs throughout the course of disease -Possibility of assessing molecular characteristics of CTCs for clinical decision-making -Could be cultured to evaluate drug resistance in vitro or in vivo and used for functional assays	-Low number in blood requires very sensitive and robust methods -High sample volume or sample enrichment approaches needed to increase likelihood of detection -Costly, specifically if background blood profiling is needed -Lacks standardization and reproducibility -Need for large-scale clinical data for validation in clinical practice
ctDNA	-Can be sampled regularly to monitor response to treatment, clonal evolution, and acquisition of resistance -Wide range of techniques for analysis available (NGS, digital droplet PCR) -Represents tumor heterogeneity (genetic alterations, level of genetic instability, number and properties of sub-clones) -Found in larger quantities in blood than CTCs -More stable than miRNA -Analysis performed in other body fluids than blood (urine, CSF)	-Concentration of ctDNA variable among patients and according to type, location, and stage of disease -Half-life of ctDNA still unclear -Source of ctDNA not clear (lytic, apoptotic tumor cells or CTCs) -Presence of background of non-altered circulating free DNA (cfDNA) from other cellular sources -Requires previous knowledge of target of interest -Need to control preanalytic aspects (rapid processing of samples to avoid cell death and release of ctDNA not reflecting tumor cells) -Need for large-scale clinical data for validation in clinical practice
miRNA	-Stable in healthy individuals (age, gender, body mass) vs altered expression in disease -Various sources (plasma, serum, urine, saliva) -Sensitive detection methods for miRNAs (sensitive biomarker) -Dynamic expression pattern associated with stage and progression of disease -Potential target for MM treatment	-Sampling methods could impact miRNA detection -Level of miRNA in patients and healthy individuals overlap (increased possibility of false negative or positive diagnosis) -Altered expression patterns of the same miRNA in various types of cancers -No single miRNA biomarker but a combination needed for clinical application
EV	-Easy to access -Present in several body fluids -Longevity and stability within circulation -Potential biomarker for early detection and prognosis of MM -Potential drug delivery vehicle and vaccine -Potential target for MM treatment	-Lack of standardization protocols -Circulating EVs can be influenced by several patient factors -Time-consuming -High cost -Heterogeneity of EV recovery population between methods -Need for correlation with clinical data

As aberrant miRNA expression is a common feature in a variety of cancers, including MM, these molecules offer exciting new opportunities for the discovery and validation of novel therapeutic targets. The published evidence on miRNA as potential biomarkers for MM and on data establishing the role of different miRNAs as prognostic factors in MM was reviewed. Whether or not specific miRNAs play a significant role in pathogenesis or treatment needs further investigation, including long-term clinical data, both in clinical trials and in real life. Some of the data on miRNAs refer to specific miRNAs found in EVs. Several studies have explored the biological function of EVs and their potential clinical use in MM patients. Because EVs can be measured through minimally invasive procedures and their contents are relevant as disease biomarkers, they have a strong potential to be translated into clinical practice [122]. As potential new biomarkers in MM, EVs may offer the advantage of assessing the cellular crosstalk between tumor cells and surrounding cells, including the potential interception of intercellular communication before onset of clinical symptoms, response to treatment, patient follow up, and disease aggressiveness. Additionally, both miRNAs and EVs constitute potential targets for therapeutic intervention. However, one should consider the current technical limitations of EV isolation, including lack of standardization protocols. Current methods for EVs isolation are still very time-consuming impairing their use in the clinical practice. More sensitive and user-friendly methods of EVs analysis such as nanoparticle flow cytometry are currently being validated and will hopefully overcome this. Additionally, new potential targets for MM diagnosis and monitoring are being evaluated. Tumor-educated blood platelets (TEPs) were recently identified as noninvasive biomarkers by sequestering EV-derived RNAs and proteins, therefore providing information on the presence, location, and molecular characteristics of cancers [123]. Platelets are fundamental components of the tumor microenvironment that mediate crucial steps in tumor progression. Takagi et al. recently demonstrated that platelets derived from MM patients are highly activated and correlated with disease status. Their work showed that platelet-mediated upregulation of IL-1β through induction of IL-6, a growth and survival factor for MM cells, is critical for MM proliferation [124]. These preclinical data suggest that not only TEP RNA could complement biomarkers used for liquid biopsy diagnosis but also disease progression might be delayed by early targeting platelet-tumor interaction in MM via platelet-regulating agents.

Future studies taking into consideration larger cohorts of patients, different disease stages, and various therapeutic settings are required to further explore the relevance of liquid biopsies in MM, both in the clinical and investigational settings.
