# Correction to: Liquid biopsies for multiple myeloma in a time of precision medicine

**DOI:** 10.1007/s00109-024-02500-1

**Published:** 2024-11-12

**Authors:** Bruna Ferreira, Joana Caetano, Filipa Barahona, Raquel Lopes, Emilie Carneiro, Bruno Costa-Silva, Cristina João

**Affiliations:** 1 Myeloma and Lymphoma Research Programme, Champalimaud Centre for the Unknown, Lisbon, Portugal; 2 Hemato-Oncology Unit, Myeloma and Lymphoma Research Programme, Champalimaud Centre for the Unknown, Lisbon, Portugal; 3 Systems Oncology Group, Champalimaud Centre for the Unknown, Lisbon, Portugal; 4 Hemato-Oncology Unit, Myeloma and Lymphoma Research Programme, Nova Medical School, Champalimaud Centre for the Unknown, Lisbon, Portugal

**Correction to: Journal of Molecular Medicine (2020) 98:513–525**

10.1007/s00109-020-01897-9

We mistakenly forgot to mention the funding for this paper.

Funding This research was funded by the Fundação para a Ciência e Tecnologia – FCT (Research Grant PTDC/MEC-HEM/30315/2017).

